# Incidence and Surgical Importance of Zuckerkandl's Tubercle of the Thyroid and Its Relations with Recurrent Laryngeal Nerve

**DOI:** 10.5402/2012/450589

**Published:** 2012-08-16

**Authors:** Emin Gurleyik, Gunay Gurleyik

**Affiliations:** ^1^Departments of Surgery, Medical Faculty, Duzce University, 81650 Duzce, Turkey; ^2^Departments of Surgery, Haydarpasa Numune Training and Research Hospital, 34500 Istanbul, Turkey

## Abstract

*Background*. Variations of recurrent laryngeal nerve (RLN) and Zuckerkandl's tubercle (ZT), which is posterior extension of lateral lobes, may affect safety of thyroidectomy. *Methods*. Total and hemithyroidectomy were surgical procedures in 60 and 40 patients, respectively. Surgical anatomy was studied in 87 right and 73 left lobes. Presence of ZT was noted and its incidence was determined. RLNs were identified and fully isolated. Relationship between ZT and RLN was established. *Results*. ZTs were identified in 66 (66%) patients and in 81 (51%) lobes. ZT was present in 53 (61%) right and in 28 (38%) left lobes. ZTs were bilateral in 15 (25%) of 60 total thyroidectomy cases. Smaller tubercles show the neurovascular crossing point. RLN was posterior (medial) to ZT in 76 (94%) occurrences. RLN was laying on anterior surface of ZT only in 5 (6%) instances. *Conclusions*. RLN is unusually laying lateral to ZT which is common structure in the thyroid. Lateral RLN may be more vulnerable to injury. Total thyroidectomy requires dissection of ZT adjacent to RLN. Based on unusual relations and variations, RLN should be fully isolated before excision of adjacent structures.

## 1. Introduction

A thyroid surgeon must have intimate knowledge about all anatomic variations of the gland affecting the safety of surgical operations. Emil Zuckerkandl (1849–1910), an Austrian anatomist has described many anatomical structures in the body [1, 2]. Zuckerkandl's tubercle (ZT) is defined as posterior extension of the lateral lobes composing of thyroid tissue only [3]. It should be included in the Nomina Anatomica as the “processus posterior glandulae thyroideae” described by Zuckerkandl [4]. It is classified into four groups according to size [5, 6]. The surgical importance of ZT can be summarized as (1) dissection and excision of ZT for total thyroidectomy and (2) close relationship between ZT and recurrent laryngeal nerve (RLN). The completeness of thyroidectomy requires removal of enlarged ZT which is posterolateral extension of thyroid lobes adjacent to RLN. Close relation of two structures urges careful, fine, and very close dissection around the nerve. Due to posterior location of an enlarged ZT, thyroid surgeon must be focused on its relations with inferior laryngeal nerve for safe identification of the nerve and resection of the tubercle. The presence of ZT and close association of RLN to an enlarged tubercle has been documented in many patients [7–10].

 We aim to study the presence of ZT, its relations with RLN, and variations of this relationship during surgical dissection of lateral lobes of the thyroid gland. 

## 2. Patients and Methods

A prospective study about surgical anatomy of the thyroid was conducted on 100 thyroidectomy patients between May 2009 and August 2011. Patients with reoperative surgery for treatment of recurrent goiter were not included. Total thyroidectomy and hemithyroidectomy (unilateral total lobectomy-isthmusectomy) are our procedures for the treatment of surgical diseases of the thyroid. All operations were performed by a single surgeon in order to provide a standard dissection. Main subjects of this study are the presence and incidence of ZT and its relations with RLN. After freeing and medially mobilizing lateral lobes of the thyroid gland at both sides with classical surgical approach, the inferior thyroid arteries were identified, isolated and a loop of silk suture was placed around arteries for traction. During surgical dissection of posterior plan we noted the presence of distinct and prominent ZT. With usual lateral approach RLNs were identified below the artery and fully isolated at both sides. If clearly delineated tubercle was identified at posterolateral aspect of the thyroid lobes, we studied its relations with RLN. The relationship between the RLN and the tubercle was established in our thyroidectomy patients. 

## 3. Results

**Figure 1:**
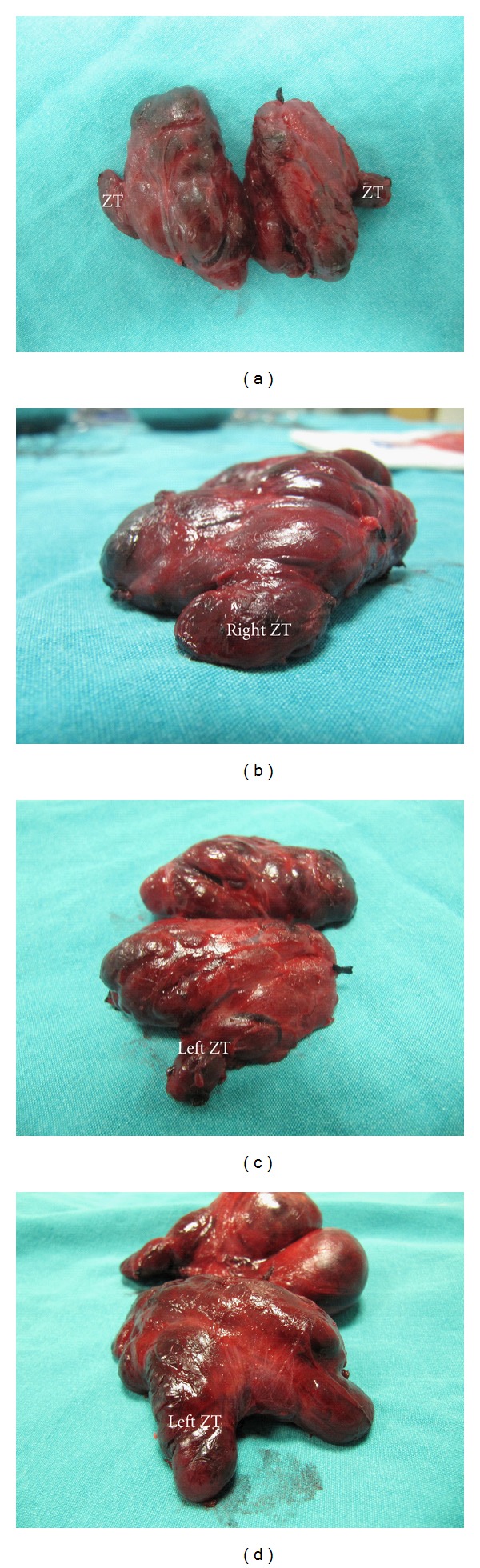
These figures show enlarged Zuckerkandl's tubercles (ZT) in the total thyroidectomy specimen. (a) Bilateral ZT, (b) right ZT, and (c) left ZT of the same patient. (d) An example of a large ZT as posterior extension of the left lobe.

Seventy-eight (78%) of our patients were women. The operation was total thyroidectomy in 60 (60%) and unilateral total lobectomy (27 right and 13 left lobes) in 40 (40%) patients. Therefore, this study was performed on 87 right and 73 left lobes of the thyroid. Larger ZTs were identified in 66 (66%) patients, and 81 of 160 (51%) excised lobes. The presence of prominent ZT was observed in 53 of 87 (61%) right and 28 of 73 (38%) left lobes. ZTs were present bilaterally in 15 (25%) of 60 total thyroidectomy cases (Table 1). Bilateral enlarged tubercles are easily observed on the pathologic specimen of the thyroid gland of the same patient (Figures 1(a), 1(b), and 1(c)). A large ZT sometimes constitutes significant part of the thyroid lobe (Figure 1(d)). After gentile lateral traction of inferior thyroid artery, RLNs have been successfully identified in all cases.

**Figure 2:**
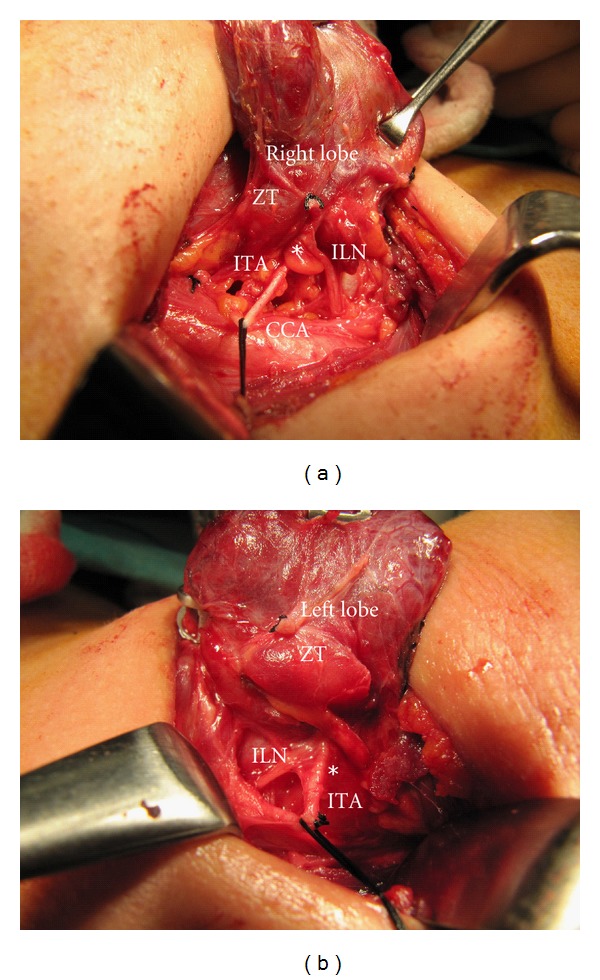
The inferior laryngeal nerve (ILN) passes posterior (medial) to the Zuckerkandl's Tubercle (ZT) near neurovascular crossing point (**∗**) with inferior thyroid artery (ITA). (a) Right side and (b) left side of two different patients.

**Figure 3:**
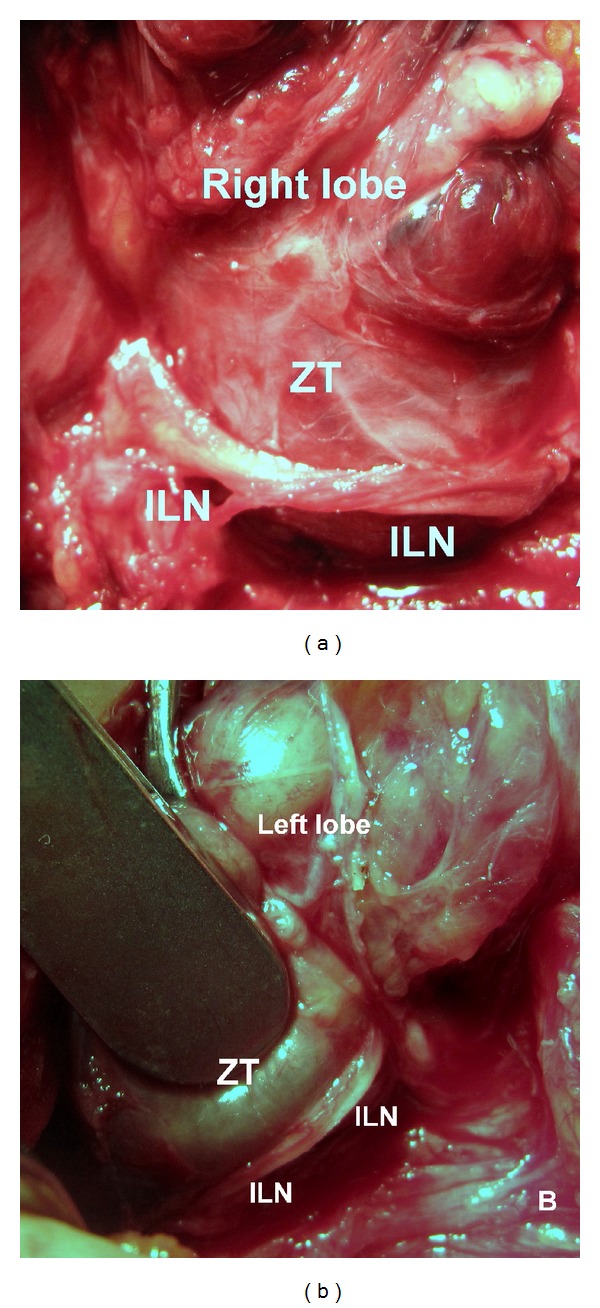
The inferior laryngeal nerve (ILN) unusually passes anterior (lateral) to the Zuckerkandl's tubercle (ZT). (a) Right side and (b) left side of two different patients.

 Results of the relationship between the tubercle and the recurrent nerve are important observations of this study. The smaller ZT was pointing like an arrow-head the neurovascular (recurrent nerve and inferior artery) crossing point in many patients. We observed common relationship between RLN and ZT in 76 (94%) of 81 tubercles; the nerve usually passes medial to the tubercle, by other words RLN is placed between the tubercle and the trachea. The nerve was located posterior to ZT (Figure 2). On the other hand, the tubercle was placed between the nerve and the trachea, pushing the recurrent nerve laterally in 5 (6%) instances. The nerve was laying on the anterior surface of ZT (Figure 3). The thyroidal tissue was totally excised (unilaterally or bilaterally) including ZT and pyramidal lobe when present without nerve injury in all patients.

## 4. Discussion

Otto Wilhelm Madelung had described in 1867 “posterior horn of the thyroid” [1, 10]. Emile Zuckerkandl has been reported in 1902 “processus posterior glandulae thyroideae” [1, 2]. The ZT is posterior extension of the gland composed of thyroidal tissue. In case of goiter formation the ZT generally enlarges synchronously at posterior site of the thyroid. First surgical importance of ZT emerges from indication of total resection of the gland that the completeness of thyroidectomy requires total removal of enlarged tubercle. Completeness of resection requires an awareness of thyroid development including attention to pyramidal remnants, to abnormalities associated with ZT [11]. Second surgical importance of the ZT arises from its relations with RLN. The resection of enlarged tubercle at posterior site of the thyroid requires delicate and careful dissection adjacent to the nerve. Identification of ZT and an understanding of the relationship between the ZT and RLN are essential for safety of thyroid operations [7, 9, 10]. Although ZT is classified into four grades according to size, this grading is mainly important for cadaveric anatomical studies. Surgeons generally perform thyroid operations on voluminous goiter that when present larger tubercles are observed on surgical specimens. Therefore, by surgical point of view an enlarged ZT parallel to goiter formation merits more interest than smaller one. It makes surgical dissection challenging at posterior site of the lateral lobes around RLN and inferior artery.

When present, mobilization of a prominent ZT has surgical importance by completeness of thyroidectomy and identification and isolation of the RLN. The presence of larger tubercles in 66% of our patients confirms that it is a common anatomical part of the gland. Many authors have recently reported the incidence of ZT as more than 50% of their patients; Kaisha et al. [12] 59%, Hisham and Lukman [7] 55%, and Gauger el al. [9] 63% (grade 3 as 45%). On the other hand, Page et al. [4] have identified ZT only in 7% of their patients. Our results of the incidence of enlarged ZT as 51% in excised lateral lobes indicate importance of the tubercle for completeness of thyroidectomy. Yalçin et al. [13, 14] have reported an incidence of grade 2 and 3 tubercles as 64% and 65% in lateral lobes. Yun et al. [10] have found grade 2 and 3 ZT in 68% (right side 72% and left side 64%) of lateral lobes. Bilateral occurrence of ZT is a less common observation as 25% in our series of total thyroidectomy cases. Gauger et al. [9] have reported (bilateral) ZT in 15% of their patients. We can comment that enlarged ZT is a common anatomical structure situated at posterolateral site of thyroidal lobes of goiter cases. Therefore, in the majority of patients the tubercle can affect completeness of thyroidectomy especially for less-experienced hands. Some brief knowledge about embryogenesis of the thyroid gland may help understanding surgical importance of ZT. The thyroid gland develops from two anlages; the larger median anlage and paired smaller lateral anlages which become attached to the posterior surface of the thyroid. It is estimated that the lateral thyroid anlage contributes perhaps 1% to 30% to the thyroid weight. Residual posterolateral projection from the lateral thyroid component is known as the ZT [1]. In the last 20 years, hemithyroidectomy and total thyroidectomy are the procedures of choice for the management of patients with goiter. In this period, the operative technique has been moved from lateral to capsular dissection. Delbridge [11] has stated that the completeness of resection has been assured by moving from an anatomically based approach to an embryologically based approach. This requires an awareness of thyroid development including attention to abnormalities associated with ZT.

RLN injury may be prevented by its full isolation based on intimate knowledge of the anatomy including all its variations [15]. Some anatomical landmarks help surgeons identifying RLN. ZT appears as an indicative arrow for the nerve and neurovascular crossing point in some patients. We can comment that after medial mobilization of the lobes, when present, ZT may be used as a landmark facilitating identification of the nerve. Many authors have previously stated that the ZT is a reliable and constant anatomical landmark as an arrow pointing the RLN [4, 5, 12–14]. The site of greatest risk during thyroidectomy to the RLN is in the last 2-3 cm extralaryngeal course of the nerve before its laryngeal entry above the trunk of the inferior thyroid artery [8]. Based on our findings ZT pointing, like an arrow head, neurovascular crossing point promotes surgeon's challenge to identify RLN. On the other hand, larger tubercle generally covers anterior surface of the nerve. Mobilization of the tubercle medially allows easy identification of the nerve at this dangerous site. Understanding the relationship between the nerve and the tubercle leads to perform safer thyroid surgery. 

 The neighboring of ZT and RLN is another important point for their relation. The resection of ZT for total thyroidectomy requires refined and meticulous dissection adjacent to the nerve. When enlarged by disease, the tubercle passes over the nerve like a bridge. This normal anatomical relationship is retained in the majority of cases [5]. Thyroidectomy technique is improved because of the constant relationship between these two structures at a level where the risk of injury is greatest [16]. Our findings in the great majority (94%) of lateral lobes with documented larger ZT confirm this statement. An enlarged tubercle usually covers a segment of the distal part of the nerve passing in the tracheo-esophageal groove. On the other hand, we also observed unusual position of ZT and RLN at both sides; RLNs are anterior (or lateral) to ZT in 6% of occurrences. Uncommon anatomical relations between RLN and ZT may affect the safety of thyroid operations. All thyroid surgeons must be aware of this uncommon variation in order to prevent injury to the nerve during total resection of the gland. Hisham and Lukman [7] have previously reported that in 6% of dissection, the RLN was on the anterior surface of the tubercle. Gauger et al. [9] have also reported that in 93% of patients with enlarged ZT, the RLN lays medial to the tubercle, and the nerve was found lateral to it in the remaining 7% of their cases. Anterior course of RLN is at highest risk of injury. The surgeon must be aware of the tubercle, and he must face the ZT without fear but with care [16]. Identification of ZT, an understanding of the relationship between the ZT and RLN, and isolation of the nerve before dissection of ZT are essential for performing safer thyroid surgery. 

 In conclusion, Zuckerkandl's tubercle which is defined as posterior extension of lateral lobes of the thyroid gland, is a common anatomical structure found in the majority of cases. Excision of the tubercle requires fine and meticulous dissection with great care because of close relationship between ZT and RLN. The RLN is uncommonly located on anterior surface of the tubercle. This unusual lateral course may increase the risk of injury. Based on the occurrence of unusual variations, we propose to identify the RLN before attempting dissection of adjacent structures. Excision of ZT is mandatory for completeness of thyroidectomy. Fine and delicate dissection with care around the ZT is also mandatory after identification and isolation of the RLN for preventing nerve injury. 

## Figures and Tables

**Table 1:** The incidence of Zuckerkandl's tubercle (ZT) in the thyroid lobes.

Operation and site	Right ZT	Left ZT	Bilateral ZT	Total
Right hemithyroidectomy	16(59)*			16 (59)
(*n* = 27)				
Left hemithyroidectomy		6 (46)		6 (46)
(*n* = 13)				
Total thyroidectomy	22 (37)	7 (12)	15 (25)	44 (73)
(*n* = 60)	37^†^ (62)	22^†^ (37)		
Right lobe (*n* = 87)	53 (67)			
Left lobe (*n* = 73)		28 (38)		

*Numbers in parentheses are percentage. ^†^Numbers includes lateral lobes of bilateral cases.
